# Supplementary material for: Pneumococcal carriage in adults aged 50 years and older in outpatient health care facility during pandemic COVID-19 in Novi Sad, Serbia
Source: PLoS One. 2022 Oct 12;17(10):e0274674. doi: 10.1371/journal.pone.0274674 (PMC9555667; doi:10.1371/journal.pone.0274674)
Supplement: S1 Appendix — (DOCX) [file pone.0274674.s001.docx]

**Appendix 1. Information leaflet for subjects**

***Pneumococcal carriage in adults ≥50 years of age*** ***in outpatient health care facility in Novi Sad***

*Streptococcus pneumoniae* (pneumococcus) is a cause of morbidity and mortality in people of all ages throughout the world. In children, *S. pneumoniae* is the most common cause of bacterial otitis media, pneumonia and bacteremia. In persons aged >65 years, the annual incidence ranged from 24 to 85 cases/100 000 population. In Europe and the United States, *S. pneumoniae* was estimated to cause approximately 30–50% of community-acquired pneumonias (CAPs) requiring hospitalization in adults. Problems are most common in persons older than 50 years of age who suffer from various chronic conditions.

It is known that mortality rates and the burden of pneumococcal pneumonia, as well as all invasive pneumococcal disease –varries between the serotypes.

To treat diseases caused by *S. pneumoniae* and to reduce the number of patients with this infection, scientists need to determine the circulating serotypes of *S. pneumoniae*. For this purpose, funded project supports research serotyping of *S. pneumoniae* in Novi Sad.

We invite you to participate in this research. In this way you can help us find the ways to protect others from infection in the future.

Your participation requires only little of your time. After discussing the details of your participation with your doctor, you must sign a document in which you declare that you agree to participate in this research. There will be no need for any additional medical procedures for this research. In order to perform certain diagnostic tests, your doctor will take nasopharyngeal and oropharingeal swab from you during your visit.

Your agreement for participation in this study is completely voluntary. You will not receive any compensation for your participation, but your contributions will be extremely valuable both for the science and for other people in society.

Your sample may be used for research of other bacterial organisms that may be significant and are not described in the project. Your data will remain confidential. Even if you agree to participate now, you can always change your mind in the future and to cancel your participation at any time, without any consequences.

Thank you in Advance!

Principal Investigator

Full. Prof. Vladimir Petrović M.D. Ph.D
