# Supplementary material for: Pneumococcal carriage in adults aged 50 years and older in outpatient health care facility during pandemic COVID-19 in Novi Sad, Serbia
Source: PLoS One. 2022 Oct 12;17(10):e0274674. doi: 10.1371/journal.pone.0274674 (PMC9555667; doi:10.1371/journal.pone.0274674)
Supplement: S2 Appendix — (DOCX) [file pone.0274674.s002.docx]

**Appendix 2. Informed Consent of the subject**

I hereby state that I am familiarized with the goals and methodology of the research, and also not to expect any kind of personal material gain.

I am aware that I can exclude from the research at any time and also if I decide to do so there will be no consequences on my treatment.

Full name and surname of participant

___________________________________

Signature

___________________________________ Date:________________

(DD/MM/YY)

**Physician:________________________________________**
