# Supplementary material for: Pneumococcal carriage in adults aged 50 years and older in outpatient health care facility during pandemic COVID-19 in Novi Sad, Serbia
Source: PLoS One. 2022 Oct 12;17(10):e0274674. doi: 10.1371/journal.pone.0274674 (PMC9555667; doi:10.1371/journal.pone.0274674)
Supplement: S3 Appendix — (DOCX) [file pone.0274674.s003.docx]

**Appendix 3: Survey questionnaire**

First and Last Name of subject: ______________________________

Date of birth: _____________________________

Gender: Male Female

Date of sampling­­­­­:___________________________

Age ________________________

1. Vaccinated with PPSV23 or PCV13 (any previous administration)? Yes No (if No, please go to question number 2)

Date of application of last dose of PPV23 or PCV13: _______________________________________

2. Contact with children aged 0-10 years in the family: 0 1 2 3 4 5 or more

3. If children aged under two years, please note if they vaccinated with PCV (if you know):______________________

4. Do your family members smoke at home? Yes No
