# Supplementary material for: Pneumococcal carriage in adults aged 50 years and older in outpatient health care facility during pandemic COVID-19 in Novi Sad, Serbia
Source: PLoS One. 2022 Oct 12;17(10):e0274674. doi: 10.1371/journal.pone.0274674 (PMC9555667; doi:10.1371/journal.pone.0274674)
Supplement: Attachment — Submitted filename: Response to Reviewers.docx [file pone.0274674.s005.docx]

**PONE-D-22-02347R1**

**Pneumococcal carriage in adults aged 50 years and older in outpatient health care facility during pandemic COVID-19 in Novi Sad, Serbia**

**PLOS ONE**

Dear Dr. Ristić,

Thank you for submitting your manuscript to PLOS ONE. After careful consideration, we feel that it has merit but does not fully meet PLOS ONE’s publication criteria as it currently stands. Therefore, we invite you to submit a revised version of the manuscript that addresses the points raised during the review process.
The manuscript has been assessed by four reviewers. Their comments are available below. Two reviewers have raised a number of concerns about the manuscript and also about the quality of the english.

Please include the following items when submitting your revised manuscript:

- A rebuttal letter that responds to each point raised by the academic editor and reviewer(s). You should upload this letter as a separate file labeled 'Response to Reviewers'.
- A marked-up copy of your manuscript that highlights changes made to the original version. You should upload this as a separate file labeled 'Revised Manuscript with Track Changes'.
- An unmarked version of your revised paper without tracked changes. You should upload this as a separate file labeled 'Manuscript'.

If you would like to make changes to your financial disclosure, please include your updated statement in your cover letter. Guidelines for resubmitting your figure files are available below the reviewer comments at the end of this letter.

If applicable, we recommend that you deposit your laboratory protocols in protocols.io to enhance the reproducibility of your results. Protocols.io assigns your protocol its own identifier (DOI) so that it can be cited independently in the future. For instructions see: <https://journals.plos.org/plosone/s/submission-guidelines#loc-laboratory-protocols>. Additionally, PLOS ONE offers an option for publishing peer-reviewed Lab Protocol articles, which describe protocols hosted on protocols.io. Read more information on sharing protocols at <https://plos.org/protocols?utm_medium=editorial-email&utm_source=authorletters&utm_campaign=protocols>.

We look forward to receiving your revised manuscript.

Kind regards,

Jose Melo-Cristino, M.D., Ph.D.
Academic Editor
PLOS ONE

***Dear Editor,***

***First of all, we would like to thank you for considering our paper for publication in your Journal. We are grateful for all the suggestions and remarks, which we find well-intended and helpful. The authors have considered all the comments and we did our best to correct the manuscript in the most appropriate and clear way. All the changes that have been made are highlighted in the manner proposed by the Journal.***

Reviewers' comments:

Reviewer's Responses to Questions

**Comments to the Author**

1. If the authors have adequately addressed your comments raised in a previous round of review and you feel that this manuscript is now acceptable for publication, you may indicate that here to bypass the “Comments to the Author” section, enter your conflict of interest statement in the “Confidential to Editor” section, and submit your "Accept" recommendation.

Reviewer #1: All comments have been addressed

Reviewer #2: All comments have been addressed

Reviewer #3: (No Response)

Reviewer #4: (No Response)

2. Is the manuscript technically sound, and do the data support the conclusions?

The manuscript must describe a technically sound piece of scientific research with data that supports the conclusions. Experiments must have been conducted rigorously, with appropriate controls, replication, and sample sizes. The conclusions must be drawn appropriately based on the data presented.

Reviewer #1: Partly

Reviewer #2: Yes

Reviewer #3: Yes

Reviewer #4: Yes

3. Has the statistical analysis been performed appropriately and rigorously?

Reviewer #1: Yes

Reviewer #2: Yes

Reviewer #3: Yes

Reviewer #4: Yes

4. Have the authors made all data underlying the findings in their manuscript fully available?

The [PLOS Data policy](http://www.plosone.org/static/policies.action#sharing) requires authors to make all data underlying the findings described in their manuscript fully available without restriction, with rare exception (please refer to the Data Availability Statement in the manuscript PDF file). The data should be provided as part of the manuscript or its supporting information, or deposited to a public repository. For example, in addition to summary statistics, the data points behind means, medians and variance measures should be available. If there are restrictions on publicly sharing data—e.g. participant privacy or use of data from a third party—those must be specified.

Reviewer #1: Yes

Reviewer #2: Yes

Reviewer #3: Yes

Reviewer #4: Yes

5. Is the manuscript presented in an intelligible fashion and written in standard English?

PLOS ONE does not copyedit accepted manuscripts, so the language in submitted articles must be clear, correct, and unambiguous. Any typographical or grammatical errors should be corrected at revision, so please note any specific errors here.

Reviewer #1: Yes

Reviewer #2: Yes

Reviewer #3: Yes

Reviewer #4: Yes

6. Review Comments to the Author

**Response to Reviewers**

**IMPORTANT!**

Comments to the Author are shown in regular fonts, while the comments to the Reviewers and the academic Editor are in the italic fonts.

Reviewer #1: The manuscript by Ristic and colleagues has been significantly improved, regarding both clarity and scientific rigor. The statistical analysis has been improved and the presentation of the results is clearer. Although the main limitation remains, the effort made by the authors to explain the rational for the study and to acknowledge its limitations is very well reflected in this version of the manuscript.

*Thank you very much for these comments. Though we strived to overcome all the limitations of our study, some of them still exist, but we sincerely hope that they have all been addressed appropriately in the present version of the manuscript.*

Reviewer #2: (No Response)

Reviewer #3: This is a revised version of the manuscript “Pneumococcal carriage in adults aged 50 years and older in outpatient health care facility during pandemic COVID-19 in Novi Sad, Serbia.
In general, the overall revision, especially for the editing of scientific language and corrections/implementations according to some requests, improved the quality of the manuscript. However, the most critical issue concerning the lack of molecular methods was not solved due to non-availability of original specimens. Authors stated that design of the study was based on the WHO recommendations (ref 18) that were published in 2014. Indeed, during the latest years a number of other studies gave indications on best procedure for pneumococcal carriage studies, especially in adults, such as “**Arguedas A, Trzciński K, O'Brien KL, Ferreira DM, Wyllie AL, Weinberger D, Danon L, Pelton SI, Azzari C, Hammitt LL, Sá-Leão R, Brandileone MC, Saha S, Suaya J, Isturiz R, Jodar L, Gessner BD. Upper respiratory tract colonization with Streptococcus pneumoniae in adults. Expert Rev Vaccines. 2020 Apr;19(4):353-366. doi: 10.1080/14760584.2020.1750378. Epub 2020 Apr 17. PMID: 32237926**”.

*Thank you for these observations. Among many others, we also carrefully read the aforementioned reference. In this publication authors highlighted that the prevalence of Spn in adults is low. They suggested that the ‘pediatric approach’ in Spn colonization determination may be insufficient in adults and that the pneumococcal detection in this population may be improved by longitudinal studies that include samples from additional respiratory sites combined with more extensive laboratory testing that include molecular techniques in addition to sensitive culture-based methods. In order to improve the surveillance of Spn in adults, authors of this study also proposed that in pneumococcal carriage studies, samples should be collect not only from nasopharynx, but also from alternative respiratory sites such as the oropharynx, saliva, or nasal wash and that the culture enrichment for pneumococcus should be used.*

*As already noted, we were not able to perform the qPCR in our study due to non-availability of original specimens. However, in order to increase the sensitivity of our study, we collected oropharyngeal swabs in parallel to nasopharingeal swabs from each participant and used culture enrcihment for Spn in all our samples, regardless of their origin. As a result, we identified two participant that harboured Spn only in their oropharynx. We would also like to point out that, despite the absence of the molecular method for further detection of our specimens, the prevalence of carriage of Spn in adults in our study was comparable with or even higher than the prevalences found in several other studies that had been performed in other countries from different geographical areas (references 3, 7, 9, 35 and 36 in our manuscript). This issue was adressed in the Discussion section of the manuscript. Thus, although we are fully aware of the limitations of our study, we do believe that our study added some important additional information that can be of interest for the wider scientific community.*

Reviewer #4: The authors have addressed most of the comments raised by the reviewers, offered mostly satisfactory responses and provided necessary information in the text, including acknowledgement of the study limitations. A few issues remain that would deserve the attention of the authors.

1) Lines 110-111. Please delete this sentence since it remains unclear, despite the authors’ previous response, how the information presented could serve to support the use or not of existing pneumococcal vaccines in adults.

*Thank you for this suggestion. We removed this sentence in the present version of the manuscript.*

2) Line 122. In English it should be “0.4%”.

*This was corrected.*

3) Lines 157-158, “The primary outcomes were colonization by Spn (culture and multiplex PCR) and Staphylococcus aureus (culture)”, please change to “The primary outcomes were colonization by Spn (culture) and Staphylococcus aureus (culture)”. The current sentence can be misleading, since it can be interpreted to mean that colonization was sough by molecular methods, which is not the case and constitutes a major limitation of the study.

*Thank you for this helpful suggestion. We changed the sentence as proposed by the Reviewer.*

4) Line 174. Typographical error, should be “gene”.

*We replaced incorrectly spelled word „gen“ with „gene“.*

5) Line 175. lytA should be in italic.

*This was corrected as suggested.*

6) Lines 179-181. Can the authors specifically state how many serotypes could have been identified by their PCR schema? This is important to interpret their “non-typeable” isolates.

*According to the CDC recommended schemes and primers for pneumococcal serotype deduction, it is possible to identify 41 different serotypes of Spn. We added this fact appropriately in the text of the present version of the manuscript.*

7) Table 1. The information under the heading “Carriage of Streptococcus pneumoniae” is redundant with that under “Swab positive for Streptococcus pneumoniae” and should be eliminated.

*As suggested, the Table 1 was corrected and the redundant data removed.*

8) Table 1. The heading “Received vaccines (PCV13 or PPSV23 )” should be changed to “Received PCV13” since no participant received PPSV23.

*Thank you for this suggestion. In the Table 1, we corrected the heading with „Received PCV13“.*

9) Lines 232-233. Please delete “with female predomination (Female vs. Male: 7 vs. 3)” since, as far as I understood, this is not a statistically supported difference.

*We agree with your suggestion. There was not statistically significant difference regarding gender, and therefore we removed this part from the sentence.*

10) Table 3. Typographical error in the heading, should read: “Non-typeable Streptococcus pneumoniae isolates”

*This was corrected as suggested.*

11) Line 255. Replace “actual” for “the current”

*The replacement was done.*

12) Line 261. Please delete “(boosted by the presence of Spn in their children)”, since this is a strange sentence construction that may be misunderstood for the fact that children themselves boost antibody production.

*We agree with your suggestion and we removed this part of the sentence.*

13) Line 276, Please replace “probably” for “possibly”.

*It was replaced.*

14) Lines 285-287, sentence starting with “The serotype distribution among our participants was not affected by the reception of vaccine (…)”. Please delete the sentence since the fact that only two participants were vaccinated precludes any conclusion regarding the effect of vaccination on carriage.

*We absolutely agree with this, so we removed this part of the sentence and accordingly rephrased the whole sentence in the present version of the manuscript.*

***Sincerely,***

***Mioljub Ristić, MD, PhD***

***Centre for Disease Control and Prevention, Institute of Public Health of Vojvodina, Novi Sad, Serbia***

***Futoška 121, Novi Sad 21 000, Serbia***

***E-mail: mioljub.ristic@mf.uns.ac.rs***
