# Supplementary material for: Pneumococcal carriage in adults aged 50 years and older in outpatient health care facility during pandemic COVID-19 in Novi Sad, Serbia
Source: PLoS One. 2022 Oct 12;17(10):e0274674. doi: 10.1371/journal.pone.0274674 (PMC9555667; doi:10.1371/journal.pone.0274674)
Supplement: Attachment — Submitted filename: Response to Reviewers.docx [file pone.0274674.s006.docx]

**PONE-D-22-02347R2**

**Pneumococcal carriage in adults aged 50 years and older in outpatient health care facility during pandemic COVID-19 in Novi Sad, Serbia**

**PLOS ONE**

Dear Dr. Ristić,

Thank you for submitting your manuscript to PLOS ONE. After careful consideration, we feel that it has merit but does not fully meet PLOS ONE’s publication criteria as it currently stands. Therefore, we invite you to submit a revised version of the manuscript that addresses the points raised during the review process.

Thank you for providing the last review of the manuscript. One important issue remains to clarify, concerning antimicrobial susceptibility. In Materials and Methods you mention that disc diffusion technique was used according to EUCAST with no further details (but including a correct reference). In Results you report one isolate resistant to amoxicillin (per os) and amoxicillin-clavulanic acid (per os) but susceptible to penicillin and to ampicillin. This result is not in accordance to the rules of EUCAST and should be corrected or further explained how the result was obtained.

A second issue (less important) is: in Table 1, values of % should have only one decimal, not two.

Please include the following items when submitting your revised manuscript:

- A rebuttal letter that responds to each point raised by the academic editor and reviewer(s). You should upload this letter as a separate file labeled 'Response to Reviewers'.
- A marked-up copy of your manuscript that highlights changes made to the original version. You should upload this as a separate file labeled 'Revised Manuscript with Track Changes'.
- An unmarked version of your revised paper without tracked changes. You should upload this as a separate file labeled 'Manuscript'.

We look forward to receiving your revised manuscript.

Kind regards,

Jose Melo-Cristino, M.D., Ph.D.
Academic Editor
PLOS ONE

**Dear Editor,**

**We would like to thank you, once again, for considering our paper for publication in your Journal. We would also like to thank you and the Reviewers for the well-intended suggestions and remarks, which helped us to improve our manuscript. We have considered them and corrected, as suggested. All the changes that have been made are highlighted in the manner proposed by the Journal. Comments to the Authors are shown in regular fonts, while the responses to the academic Editor (and the Reviewers) are in the italic fonts, as follows**:

Editor:

One important issue remains to clarify, concerning antimicrobial susceptibility. In Materials and Methods you mention that disc diffusion technique was used according to EUCAST with no further details (but including a correct reference). In Results you report one isolate resistant to amoxicillin (per os) and amoxicillin-clavulanic acid (per os) but susceptible to penicillin and to ampicillin. This result is not in accordance to the rules of EUCAST and should be corrected or further explained how the result was obtained

*Thank you for this important observation, which relates to our unintentional mistake that could be misleading. Indeed, we have determined antimicrobial susceptibility of our isolates using disc diffusion technique according to EUCAST, and found one non-typeable isolate that was resistant to amoxicillin and amoxicillin-clavulanic acid, but it was also resistant to penicillin and ampicillin, as well. However, the data on penicillin and ampicillin resistance have been inadvertently omitted from Table 4 in the original version of the manuscript, and, consequently, the susceptibility pattern of this isolate was not properly reported. Therefore, in the present version of the manuscript, we have changed Table 4 to include correct results of susceptibility of our isolates. Accordingly, the statements in the Abstract, Result section on Antimicrobial resistance patterns of Streptococcus pneumoniae and in Discussion section referring to resistance of isolates to Beta-lactam antibiotics were also changed.*

Editor:

A second issue (less important) is: in Table 1, values of % should have only one decimal, not two.

*As suggested, Table 1 has been corrected in the revised version of the manuscript and all values of percentages now contain only one decimal.*

***Sincerely,***

***Mioljub Ristić, MD, PhD***

***Centre for Disease Control and Prevention, Institute of Public Health of Vojvodina, Novi Sad, Serbia***

***Futoška 121, Novi Sad 21 000, Serbia***

***E-mail: mioljub.ristic@mf.uns.ac.rs***
